# Assessment of Anti HSV-1 Activity of Aloe Vera Gel Extract: an *In Vitro* Study

**Published:** 2016-03

**Authors:** Fahimeh Rezazadeh, Maryam Moshaverinia, Mohammad Motamedifar, Montazer Alyaseri

**Affiliations:** 1 Dept. of Oral and Maxillofacial Medicine, School of Dentistry, Shiraz University of Medical Sciences, Shiraz, Iran.; 2 Shiraz HIV/AIDS Research Center, Dept. of Bacteriology and Virology, Medical School, Shiraz University of Medical Sciences, Shiraz, Iran.; 3 Students’ Research Committee, School of Dentistry, Shiraz University of Medical Sciences, Shiraz, Iran.

**Keywords:** Aloe vera, Herpes Simplex Virus-1, Herbal Medicine, Antiviral, Cell Culture

## Abstract

**Statement of the Problem:**

Herpes simplex virus (HSV) infection is one of the most common and debilitating oral diseases; yet, there is no standard topical treatment to control it. The extract of Aloe vera leaves has been previously reported to have anti-inflammatory, antibacterial, and also antiviral effects. There is no data on anti-Herpes simplex virus type 1 (HSV-1) activity of Aloe vera gel.

**Purpose:**

This study aimed to evaluate the anti-HSV-1 activity of Aloe vera gel in Vero cell line.

**Materials and Method:**

In this study, gel extraction and cytotoxicity of various increasing concentrations of Aloe vera gel (0.2, 0.5, 1, 2, and 5%) was evaluated in Dulbecco’s Modified Eagle Medium (DMEM) containing 2% fetal bovine serum (FBS). Having been washed with phosphate buffered saline, 50 plaque-forming units (PFU) of HSV-1 was added to each well. After 1 hour of incubation at 37°C, cell monolayers in 24 well plates were exposed to different increasing concentrations of Aloe vera gel. The anti-HSV-1 activity of Aloe vera gel in different concentrations was assessed by plaque reduction assays. Data were analyzed by using One-way ANOVA.

**Results:**

The cytotoxicity assay showed that Aloe vera in prearranged concentrations was cell-compatible. The inhibitory effect of various concentrations of Aloe vera was observed one hour after the Vero cell was infected with HSV-1. However, there was no significant difference between two serial concentrations (*p*> 0.05). One-way ANOVA also revealed no significant difference between the groups. The findings indicated a dose-dependent antiviral effect of Aloe vera.

**Conclusion:**

The findings showed significant inhibitory effect of 0.2-5% Aloe vera gel on HSV-1 growth in Vero cell line. Therefore, this gel could be a useful topical treatment for oral HSV-1 infections without any significant toxicity.

## Introduction

Herpes simplex is the most common infectious virus in human. Herpes simplex virus type 1 (HSV-1) is an enveloped virus that causes primary gingivostomatitis, herpes labialis, and other important infections such as encephalitis, ocular and genital infection, meningitis, and pneumonitis.[1-3]

Therapeutic choice for these infections includes the use of topical or systemic antiviral agent. Acyclovir, as a gold standard and effective agent against herpes simplex virus (HSV), is widely used for treatment of herpetic infection. However, increasing resistance to acyclovir during long-term medication, especially in immunocompromised patients, and also its nephrotoxicity are the major concerns in the management of such infections.[4-5] Additionally, it is not effective for the treatment of latent infections.[1]

Topical agents have possible advantages, but do not penetrate effectively and, therefore, have only negligible benefit in treatment.[6] Moreover, frequent and recurrent use could be expensive for the patients. Based on these reasons, introducing new natural antiviral compounds is increasing. Interest in employing these agents has been enhanced by investigators and clients due to preference for natural medicines and concerns about toxic effects of synthetic materials.[2-3] Remedial plants have been traditionally used for the treatment of diseases with less cost and more efficacies.[4, 7-8] World Health Organization (WHO) reported that about 80% of world population use medicinal plants to treat the disease.[9]

Aloe vera is a stem-less plant from the Lily family. It is native for dry and hot countries and has been used medicinally for over thousands of years by Egyptian, Indians, Chinese, and other Asian cultures. Its thick and fleshy green leaves contain a clear, soft, moist, and smooth mucilaginous fluid that is referred to as Aloe vera gel.[10] This gel contains over 75 active ingredients that has been declared to have anti-inflammatory, anti-oxidant, immune improving, anti-cancer, healing, and anti-ageing materials.[11]

This herbal medicine has been reported to have inhibitory effects against some kind of viruses such as human cytomegalovirus, herpes simplex virus type 2 (HSV-2), and poliovirus.[1] Previous studies also showed its antifungal, antiviral, and antibacterial activity.[1, 7] Its topical form is used to improve healing of wounds.[1]However, to the best of the authors’ knowledge, no similar scientific study has been performed on the anti-HSV-1 activity of Aloe vera gel. Since herpetic infections remain a universal health care problem and there is no topical treatment for control of recurrent intraoral herpes, finding herbal medicine with an experimentally-proven anti-herpetic effect and little adverse effects can be useful for prevention and treatment of these lesions . So, this study attempted to investigate the antiviral effects of Aloe vera gel extract on HSV-1. 

## Materials and Method

The present study was designed to evaluate the antiviral activity of Aloe Vera by using its gel extract. The study was performed in Virology Department, Shiraz University of Medical Science, Shiraz, Iran. In this study, the antiviral activity of Aloe Vera was assessed by plaque reduction assay after gel extraction and cytotoxicity evaluation. 

**Preparation of Aloe vera extract**

Leaves of Aloe vera were collected from a farm around Shiraz, Iran. It was confirmed by an expert from the Department of Biology, Shiraz University. Mature, healthy and fresh leaves of Aloe vera, approximately 90-100 cm long were washed with fresh water for 5 min and rinsed with sterile distilled water. Their thick epidermis was removed and they were cut transversely into pieces. Then the colorless solid mucilaginous thick tissue (Aloe vera gel) was scraped out by using a sterile knife and collected in a sterile container. One hundred grams of the gel was mixed in one liter of 2% dimethyl sulfoxide (DMSO) and kept at 4°C to be used as a solution. Then, it was sterilized by filtration.

**Virus Stock**

HSV-1 was isolated from the lip lesions of a patient and was confirmed by neutralization test using guinea pig anti-HSV-1 serum (NIH; USA).

**Cell Culture and Cytotoxicity Assays**

Vero cells line (Cell Bank of Pasteur Institute; Tehran, Iran) was prepared for assessing cytopathic effect of HSV. In order to prepare that, confluent Vero cells were grown in Dulbecco’s Modified Eagle’s Medium (DMEM) (Sigma; USA) containing 5% fetal bovine serum (FBS) (Gibco; Germany), 0.14% sodium bicarbonate, 100 U/mL penicillin, 100 mg/mL streptomycin sulfate, and 0.25 g/mL amphotericin B.

Grown Vero cell monolayers in sterile 24-well plates (NUNC; Denmark) were washed twice with phosphate buffered saline (PBS). Increasing concentrations of Aloe vera (0.005, 0.01, 0.02, 0.05, 0.1, 0.2, 0.5, 1, 2, 5%) were added to each well. plates were incubated at 37°C with atmosphere of 5% CO_2_ for 72 hours. After being rinsed with PBS, 50 µL working solution of (Microculture Tetrazolium Test) MTT was added to each well; 50 µL DMSO solutions was added to the well after 2 hours of incubation in 5% CO_2_. After 15-30 minutes of incubation with plate shaking, 96-well plates were placed in Enzyme-Linked Immunosorbent Assay (ELISA) plate reader and light absorption was measured in 450 nm for each well. 

**Antiviral Effect Assays**

Various increasing concentrations of Aloe vera gel (0.2, 0.5, 1, 2, and 5%) were prepared in DMEM containing 2% FBS (maintenance medium). After washing with PBS, 50 plaque-forming units (PFU) of HSV-1 were added to each well. They were incubated at 37°C for 1 hour; then, the cell monolayers in 24-well plates (NUNC; Denmark) were exposed to different increasing concentrations of Aloe vera extracted gel. 

**Figure 1:**
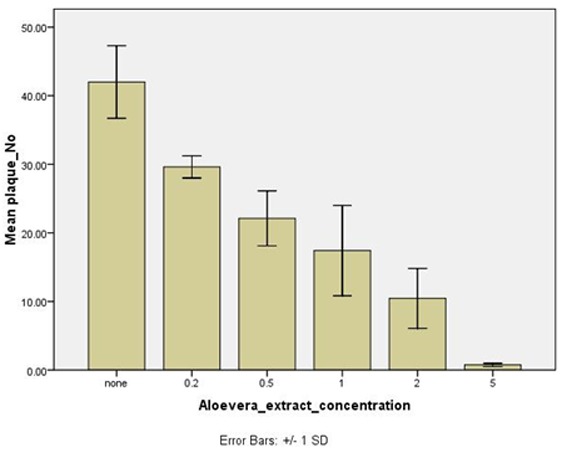
Growth of HSV-1 (PFU) in the absence (control) and presence of various concentrations of Aloe vera gel extract. The data are from three experiments in quadruplicate.

Controls for each series of experiment included uninfected cell culture with or without the extract and also normal cell monolayers without exposure to the extracts and inoculated with the virus. Following the incubation period at 37°C and 5% CO_2_ for four days, the contents of each series of wells were removed and virus infectivity was determined by using the direct plaque assay. The virus plaques formed on cells were fixed with methanol for 10 minutes and stained with 0.5% crystal violet solution; then, they were counted. (Figure 1) Experiments were performed three times in quadruplicates.

**Statistical Analysis**

The anti-herpetic effects of Aloe vera in different concentrations were compared with one another and with the untreated control group by using One-way ANOVA. SPSS software, version 17, was employed for the statistical analysis. Duncan's test was also performed as the post-hoc test. *p*< 0.05 was considered statistically significant. 

## Results

**Cytotoxicity of Aloe vera gel**

No toxicity was detected for Vero cells in all of the studied concentrations of Aloe vera gel compared with the untreated healthy cells. Based on the results, up to 5% of the gel extract was nontoxic for Vero cells. 

**Anti–HSV-1 effect of Aloe vera gel**

**Figure 2:**
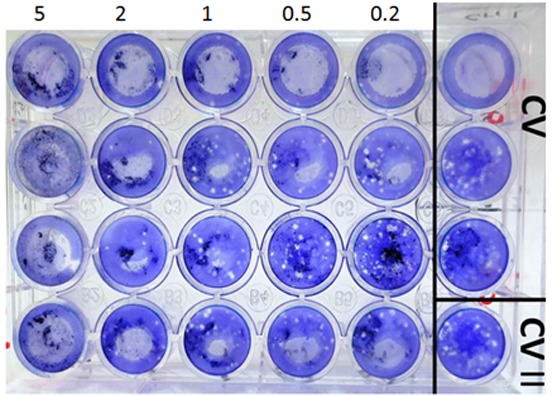
Reduction of HSV-1 plaques by Aloe vera gel extract. The effect of Aloe vera gel extracts at the concentrations of 5, 2, 1, 0.5, 0.2%, in vertical rows showing reduction of viral plaques in concentration-dependent manner compared to the control (CV) wells.

All concentrations of Aloe vera gel (ranged from 0.2 -5%) significantly inhibited the HSV-1 replication (*p*< 0.05) when applied after infection of Vero cells with HSV-1 (Figure 1). As indicated in Figure 2, the number of HSV-1 plaques was significantly reduced by different concentrations of Aloe vera gel compared with the control HSV-1 infected Vero cells without Aloe vera. One-way ANOVA showed that different concentrations had significantly different antiviral effects (*p*= 0.0001). However, based on post-hoc analysis, antiviral effect of gel at 0.2 and 0.5% concentrations was not significantly different (*p*= 0.32). Higher concentrations of Aloe vera gel (1, 2, and 5%) had significantly more anti-viral activity than 0.2% and 0.5% concentrations (*p*< 0.05). Aloe vera extract at concentration of 5% had the maximum effect on reduction of virus plaques when presented after virus infection of the cells; however, the antiviral effect of this concentration was not different from 2% concentration (*p*= 0.12). Concentrations of 1, 0.5, and 0.2% of Aloe vera gel had significantly less anti-HSV-1 activity than 5% (*p*≤ 0.005).

## Discussion

Herpes simplex virus infection is one of the most common and debilitating oral diseases; however, no standard topical treatment is known to control it. Herbal medicine has a long history for the treatment of various oral diseases. The study of antiviral activity of different herbal extract is in the viewpoint of several investigators. Aloe vera is one of these plants and based on the previous studies, the gel extracted from Aloe vera leaves has anti-inflammatory, anti-bacterial, and antifungal properties.[7, 12] There is also a report on the antiviral properties of Aloe vera against HSV-2[1] however; there is no data on anti-HSV-1 activity of Aloe vera gel. So, the current study was designed to evaluate the anti-HSV-1 activity of Aloe vera gel in cell culture system. In this study, HSV-1 was selected because its infection is one of the common viral diseases and acyclovir resistance is increasing, especially in post-transplant patients.[13] Drug toxicity is also important in patients with kidney disease.[2] To the best of our knowledge, this is the first study about the anti HSV-1 activity of extract of Aloe vera which is grown in South of Iran. 

The Aloe vera gel contains about 99% water and 0.5–1% solid material including vitamins, minerals, enzymes, polysaccharides, polyphenols, sterols, indoles, phenolic and organic acids. Many of the remedial effects of Aloe vera leaves extract are attributed to the polysaccharides of the parenchyma of leave, but it is believed that most biological activities take place due to the synergistic action of all its chemical materials.[10] Other active components of the Aloe vera gel with health benefits are acetylated mannans, polymannans, anthraquinone C-glycosides, anthrones and anthraquinones, as well as lectins.[9, 14]

This study examined the potential cytotoxicity of the Aloe vera gel extract. Results of the cytotoxicity analysis indicated that none of the tested concentrations of Aloe vera gel (up to 5%) was cytotoxic for Vero cells. This observation is in agreement with Sydiskis *et al.*,[15] and in contrast with Zandi *et al.*’s report.[1] This difference could be due to the type of extract (glycerin extract versus DMSO extract) or the species of the plant which was used in the study. The other reasons for the difference between the cytotoxicity results may be related to the varieties in exposure times, viable cell assay tests, and the cell culture conditions and reagents. In the current study, purified extract of Aloe vera gel was prepared and DMSO was used as the solvent. The glycerin extract, as previously evaluated, had toxic effect on the cultured cells.[1]

In order to determine the Aloe vera anti-HSV-1 activity after cell infection, Vero cells were treated with Aloe vera gel extracts after HSV-1 adsorption. Then, *in vitro* antiviral effect of Aloe vera gel extract was evaluated by plaque reduction assays. Our results indicated significant anti-herpetic effect after virus inoculation. It may show the mode of antiviral activity to be similar to that of anti-herpes virus drugs such as acyclovir. Thus, Aloe vera gel may be suitable as a remedial agent against HSV-1 growth and shedding. This study detected significant inhibitory effect of 0.2-5% Aloe vera gel on HSV-1 growth in Vero cell line. This finding was in agreement with another study that reported the antiviral effect of glycerin Aloe vera extracts on HSV-2 and Influenza-A virus.[1, 16]

All the five concentrations of 0.2, 0.5, 1, 2, and 5% of gel extracts revealed varying degrees of inhibitory effect against the HSV-1. (Figure 1) The highest concentration (5%) of gel extract of Aloe vera displayed maximum of antiviral effect. This study showed that Aloe vera extract may provide the advantage of inhibition of HSV-1 growth as was seen even at low concentration of 0.2%, as well. It should be noted that we considered acyclovir as a well-known anti-herpetic drug as positive control and no plaques was produced in 1250 µg/mL of it. Although in such low concentration of acyclovir, antiviral efficacy may be higher than that for Aloe vera gel, continual of increasing resistance to acyclovir in addition to its side effects are the major concerns which prompt us to rely on alternatives like Aloe vera gel for the management of HSV infections.

This inhibitory effect of Aloe vera against HSV-1 may be due to polysaccharides, emodin, or anthraquinone.[1] Emodin was shown to have inhibitory effect on replication of enveloped viruses. Small molecules in different plants including phenolics and polyphenols were reported to be as active as anti-herpetic agents;[8, 17-18]they are also found in Aloe vera gel. In addition, other components such as emodin, chrysophanic acid and hypericin demonstrated antiviral activities against hepatitis B virus and poliovirus.[16, 18]

Based on the results of the present study, considering the potential of Aloe vera gel as a candidate for anti-herpetic mouthwash or oral gel, consumption of this gel can be suggested to decrease the viral contamination of saliva and also to reduce the infectivity and duration of herpetic ulcers, especially in refractory lesions. 

Hence, Aloe vera might be considered as a new option for treating resistant HSV-1 lesions without the adverse effects of standard treatment. Moreover, further studies with more precise molecular methods are recommended for in vivo assessment of the biologic activity of Aloe vera gels. Other studies with different extracts of Aloe vera are also recommended for antiviral evaluation. 

The results of two studies that evaluated antibacterial and antifungal activity of Aloe vera showed that the acetone extract was significantly more effective than ethanol and aqueous extracts.[12, 14] In another study, methanol extract had more antibacterial activity than ethanol and distilled water extracts of Aloe Vera leaves.[9]

## Conclusion

The present study revealed the possibility of presence of some bioactive components in Aloe vera gel with anti-HSV-1 activity without any significant toxic effect in concentrations of 0.2-5%. The findings also suggest that Aloe vera gel can be a useful topical treatment for oral HSV-1 infections. This study anticipated to present a new and effective herbal drug for the treatment of oral herpes disease. 
